# Modern Dimensional Analysis Involved in Polymers Additive Manufacturing Optimization

**DOI:** 10.3390/polym14193995

**Published:** 2022-09-23

**Authors:** Zsolt Asztalos, Ioan Száva, Sorin Vlase, Renáta-Ildikó Száva

**Affiliations:** 1 Department of Mechanical Engineering, Transilvania University of Brasov, B-dul Eroilor, 29, 500036 Brasov, Romania; 2 Romanian Academy of Technical Sciences, B-dul Victoriei, 030167 Bucharest, Romania

**Keywords:** polymers, additive manufacturing, 3D printing, dimensional analysis

## Abstract

The paper aims to use Modern Dimensional Analysis (*MDA*) to study the polymers additive manufacturing optimization. The original part of the work is represented by the application of this nonconventional method in the field of polymers additive manufacturing. The laws of the model provide the complete sets of dimensionless variables, which cannot be offered by any of the classical methods (such as Geometric Analogy, Theory of Similarity, and Classical Dimensional Analysis). The validation of the method was performed experimentally. The original part of the work is represented by the application of this nonconventional method in the field of polymers additive manufacturing optimization. An application is presented and the necessary steps are analyzed one by one.

## 1. Introduction

Dimensional analysis is a method used since the beginning of the development of sciences to analyze the relationships that exist between different physical quantities. With the help of this method, the correctness of some relationships proposed by different researchers was checked, from a logical point of view, but it also allowed obtaining some dependency relationships between different physical quantities. Historically, it is difficult to determine the precise moment of the launch of this theory, but the concept of physical dimension, which is the basis of dimensional analysis, was introduced by Fourier in 1822 [1]. In the beginning, the method was mainly used for verifications and research on the plausibility of some hypotheses introduced by different researchers in their works. However, with the development of the technique, the field of applicability of this method has diversified a lot, and the engineering applications have caused the fields in which it proved its usefulness to be very varied [2]. The mathematical and logical bases of dimensional analysis are presented in many works. I mentioned some of them [3,4,5,6]. Dimensional Analysis can be used to formulate pertinent hypotheses about physical phenomena that can then be verified experimentally or using more advanced models [7,8,9]. At the same time, the specificity of the various possible applications forced the researchers to develop the method and improve it, to be able to obtain relevant results, in a short time and with minimal costs [3,10,11,12,13,14,15]. All these results led to the formulation of the Modern Dimensional Analysis, which better covers all cases that may appear in engineering practice [16,17,18].

The study of complex phenomena on models (usually reduced to scale) instead of prototypes has become a widespread practice in the field of engineering due to the simplicity, lower cost price and to the repeatability of experimental investigations under rigorously controllable conditions and with less numerous qualified personnel. The first approaches to the model–prototype correlation were aimed at the implementation of the Geometric Analogy, where through the proportionality of the dimensions and the equality of the angles, homologous points, homologous lines, homologous surfaces, homologous volumes of the model and the prototype could be defined, respectively [19]. Later, by means of the Theory of Similarity, the sphere of comparison also extended to some problems of functional similarity, establishing similar processes, which take place both in the prototype and in the model, carried out in homologous points and in homologous times in the two structures.

Another means, more efficient, but not ideal, is the Classical Dimensional Analysis (CDA) [20,21], when dimensionless groups *π_j_* will be constituted, the number of which is obtained by applying Buckingham’s theorem. However, there are also a number of shortcomings, such as: the rather arbitrary and difficult way of establishing dimensionless groups; imposing deep knowledge of the basic physical phenomenon; the complete sets of these dimensionless groups *π_j_* can only be obtained in certain special cases. An effective solution is offered by the version developed by the author of the works [16,17], hereinafter referred to as Modern Dimensional Analysis (*MDA*). *MDA* provides an original, unitary, easy and efficient strategy in determining complete sets of dimensionless groups *π_j_*. The complete set of the Model Law (*ML*) helps us to establish (depending on the dimensions adopted a priori for the prototype) the dimensions of the model, respectively, for the sought dependent variable of the prototype: its predictable size according to the results of the measurements made on the model [22,23]. Another great advantage of the *MDA* lies in the fact that if a variable is insignificant (irrelevant), either from a physical or dimensional point of view, it can be eliminated, without the *ML* undergoing significant changes (of essence) regarding the characterization of the studied phenomenon. Another important aspect is that the set of relationships contained in the *ML* do not represent calculation relationships in the usual sense, but only firm correlations, established between the scale factors of the variables involved in the description of the analyzed phenomenon [24,25]. A number of additional applications are presented in references [26,27,28].

Dimensional analysis has been developed and applied in numerous fields, where it has proven its usefulness and quasi-universal applicability in Civil Engineering [29,30,31,32], Aeronautical Science [33,34], Automotive Engineering [35,36,37], Mechanical Engineering [38,39], Medicine [40,41,42] and other interesting applications.

The use of polymer composites as raw material in additive manufacturing (*AM*) allowed a robust production and the production of parts with better mechanical properties than by using unreinforced polymers. Following the studies carried out, it was found that in general, *AM* available commercially can benefit from the advantage offered by reinforced fibers through different techniques [43].

Fiber-reinforced polymers could also be used for 4D printers to control shape change after 3D printing. There are also problems created by the use of these materials such as sleeping of voids, poor adhesion between the fibers and the matrix, blocking of the printer due to the inclusion of filler, increased curing time, and modeling and simulation being made much more difficult. The works in the field try to solve these mentioned problems [44,45,46,47].

The advantages and disadvantages of the procedure are presented in [48]. Comparisons are made with other technologies such as injection molding or the use of cutting machines. There is very wide use of the method, and achievements in the field of electronics, aerospace engineering and biomedical engineering are highlighted [49,50,51,52,53]. Important benefits and limitations are identified to clarify and motivate future work in this area [54,55].

In parallel with the manufacturing, aspects of the design, the additives used and the processing parameters must be addressed in relation to increasing the construction speed and improving the precision, functionality, surface finish, stability, mechanical properties and porosity [56,57]. Applications are currently being developed in light engineering, architecture, food processing, optics, energy technology, dentistry, drug delivery and personalized medicine [58,59,60]. Recent research in the field can be found in [61,62].

The modern approach to the problem of real structures Is based on establishing as precise correlations as possible between their behavior and those of scaled structures. The initial structure is called the prototype, and the one made to scale (usually reduced scale) is the model. Based on the above-mentioned references, the following shortcomings can be mentioned among others:

- The Geometric Analogy can only be applied with the strict observance of the existence of well-defined ratios between all dimensions (proportions) of the compared structures (prototype and model) or in the case of a very small number of variables;
- Similitude Theory already operates with dimensionless variables, but their number is relatively small, and their identification method requires solid knowledge in the field;
- *CDA* widely uses dimensionless variables, also called quantities $\pi_{j},\;\;j=1,\;\ldots ,\;n$, but with certain shortcomings, such as [63,64,65,66,67,68,69]: their establishment is non-unitary, sometimes even chaotic, and depends mainly on the ingenuity of the one who applies it; *CDA* requires solid/deep knowledge in the field, both for choosing the most eloquent analytical relationships in describing the analyzed phenomenon, and in grouping the terms from these relationships, in order to establish the desired dimensionless variables; only in special cases it allows highlighting the complete set of dimensionless variables and consequently the Model Law that will result from them; it is not an easy and accessible method for the average researcher, being especially a method intended for established theoreticians.

However, in a series of works, these principles of Geometric Analogy, Similitude Theory, and *CDA*, are applied to thermal phenomena [70,71,72] and to aspects of engineering [41,73,74], respectively. Many researches have presented the advantages of Modern Dimensional Analysis (*MDA*)applied to this type of problems [75,76,77,78,79,80,81,82,83,84].

Faced with these shortcomings, the improved method, hereinafter referred to as *MDA*, [16,17,85,86,87,88,89,90,91], comes to offer a solution, which practically eliminates all these shortcomings, becoming a unitary, safe and particularly accessible method for any researcher. The net advantages of *MDA*, successfully applied over the years and by the authors in the works [18,22,23,26,28,92,93], were presented in other work [16,17,22,26].

The application of *MDA* principles to new phenomena, such as the one analyzed below, proves once again that it represents a method with indisputable perspectives, therefore of the future, for a wide range of aspects of engineering.

In the desire to increase savings, cost price, material, labor, etc., the reduced-scale models, on which the expected qualities of the respective product can be tested in advance (i.e., the prototype), becomes a desire. Therefore, *MDA*, which provides important information on the full-scale part (prototype) through thorough experimental investigations on models (parts made to a certain scale, favorably chosen), complements and improves *AM* technology. Thus, for example, if it is desired to obtain a relatively voluminous piece of plastic material with certain qualities (stiffness, weight, bearing capacity, etc.), then it becomes useful to test these qualities on a model, which by means of firm correlations with the initial piece (which at *MDA* is the Model Law) will be able to ensure this desired response of the prototype. 

The advantages of combining *AM* with *MDA* becomes even more eloquent in the case of parts made of two or three different materials, some of which have the role of ensuring wear resistance, others rigidity or resistance to chemical agents, weathering, etc.

This is why the authors initiated this somewhat unusual approach to *AM* through the lens of *MDA*. They express the hope that these results/investigations can and will be extended to obtain the maximum benefits of *AM* technology. *AM* has the advantage of allowing the manufacture of customized objects on demand, thus avoiding the storage of parts. There is currently a continuous transition from rapid prototyping to rapid manufacturing. This brings new challenges for engineers. From this brief analysis, one can highlight (even if only partially) the flexibility offered by *MDA* over *CDA* and the rest of the methods that use correlation between prototype and model.

## 2. Model Law for Polymers Beams

**Figure 1:**
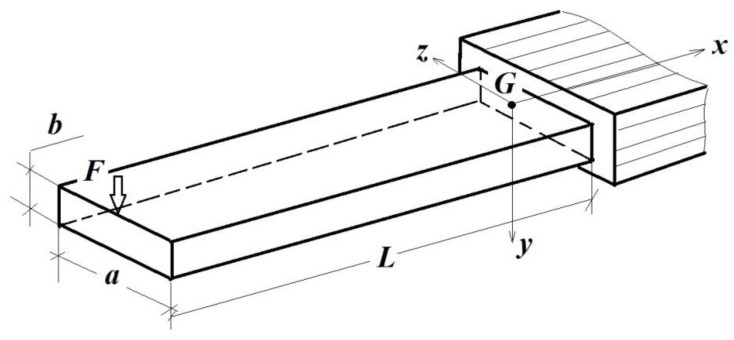
The testing beam.

**Figure 2:**
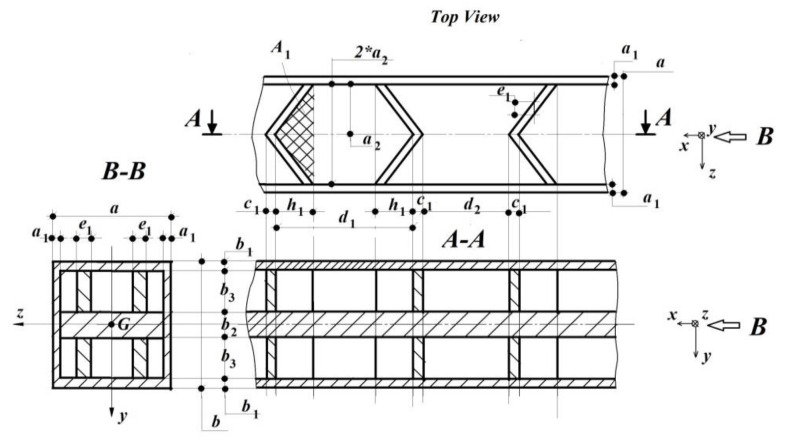
A possible version of filling the volume of the beam.

In order to develop and validate the *ML* in the case of these additive technologies through 3D printing, the authors started from the case of the beam (cantilever) subjected to simple bending. In Figure 1, the dimensions a [m],  b[m],  L[m], the reference system xGyz and the applied force F [N] are given. In Figure 2, compared to the options offered by the software of 3D printers with the help of which the element is obtained by addition, another way of filling the nominal volume of the part is presented, namely, with the help of ribs, located on two levels, in a desired order and imposed by the desired mechanical response of that part.

There are a number of detailed studies in which different researchers have investigated basic aspects of the influence of direction of filament deposition, type of filling degree, deposition speed, etc., on obtaining an optimal final product. This optimum refers not only to the achievement of certain mechanical properties of the final product, such as maximum rigidity, minimum weight, minimum degree of wear, resistance to the action of the environment, the weather or corrosive agents, but also to reduce manufacturing time, competitive shapes and design, etc. Modern machines (3D printers) are provided with particularly complex software, which allows the provision of some parameters, which will precisely ensure the achievement of the previously mentioned desires, not only by arranging the filament according to a certain geometry, but also by combining two or more types of filaments (either all plastic, or even some metallic). Thus, for example, it becomes possible to obtain molds with a metallic interior and the rest from plastic material, necessary for the manufacture of unique spare parts at a particularly advantageous cost price, without waste and with minimal time consumption.

In order to illustrate the flexibility of the *MDA*, regarding the cross-sections, which can be modelled, a relatively complex example was given, where in addition to reducing the volume of the final piece, it was also desired to preserve a certain degree of rigidity; these sections can be easily made with current 3D printers, but modelling them using other methods (Geometric Analogy, Similarity Theory or Classical Dimensional Analysis) is very unlikely; *MDA* in this regard provides the facilities required by a competitive *AM* industry.

The arrangement of these ribs, although at first sight chosen arbitrarily, leads to an optimal structure from the point of view of a beam (a bar subjected to bending). Obviously, depending on the main demands of the elements of the new product, achievable through *AM* technology, these ribs will have different layouts, which a good engineer, with adequate knowledge in the field of Material Strength and Elasticity Theory, will notice and impose in their configuration (of the disposition of the nerves). In this sense, even a preliminary numerical analysis can be of great use to the less initiated in order to highlight/identify the location of the isostatic lines (lines of maximum stress, along which the principal normal stresses are arranged), along which to arrange these ribs. As is well known, these principal normal stresses are either tensile or compressive, and the shape of the cross-section of the rib which is to take them will differ accordingly.

**Figure 3:**
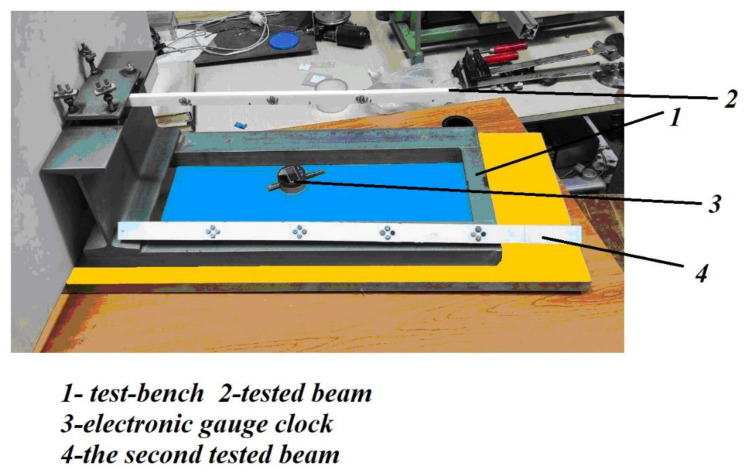
The experimental setup.

Figure 3 shows the two bars, considered as prototype and model. The plastic material used (PLA-Polylactic Acid), based on data from the literature, respectively, from the manufacturing company, had the modulus of elasticity of

$$E=E_{1}=E_{2}=2.31\cdot 10^{9}\;\frac{N}{m^{2}}\;.$$

and the scale factor results:

$$S_{E}=\frac{E_{2}}{E_{1}}=1\;.$$

From the sixth element of the Model Law, that is, from

$$\pi_{6}=F\cdot L^{3}\cdot {\left(E\cdot I_{z}\right)}^{-1}=1\;\Rightarrow \;S_{F}=\frac{S_{EIz}}{{\left(S_{L}\right)}^{3}}$$

the magnitude of force *F*_2_ will result, according to relation (18).

In order to obtain higher stiffnesses, the two beams were rotated by 90° from the position in Figure 1. Thus, even if the applied forces were relatively high, the displacements obtained were within acceptable limits. After applying the force *F*_2_ to the model, the vertical displacement at the free end of the bar *v_2_* was measured.

From element no. 1 of the *ML*, i.e., from

$$\pi_{1}=v\cdot L^{-1}\cdot {\left(E\cdot I_{z}\right)}^{0}=1\;\Rightarrow \;S_{v}=S_{L}$$

resulted in the predicted displacement value *v*_1_ for the prototype. In order to verify the correctness of this quantity, a load with the predicted force *F*_1_ was carried out on the prototype, obtaining a value *v*_1*M*_, which differed only by 1.08% from that predicted by the *ML*. Thus, the *ML* was found to be valid.

Based on those summarized in the works [16,17,18,22,23,26,28,92,93], the deduction of the Model Law involves the following basic steps:

1. The choice of variables, which can influence to a certain extent the analyzed phenomenon; here is the vertical displacement v [m] of the beam at the level of the applied force F [N], namely:
  - The dimensions of the beam a [m],  b[m],  L[m], but in the general case the area defined by the ribs and, respectively:
    (1) $$A_{1}\;\left[m^{2}\right]=\frac{2\cdot a_{2}\cdot h_{1}}{2}=a_{2}\cdot h_{1}\;;$$
  - The applied force F [N];
  - Longitudinal modulus of elasticity (Young) $E\;\left[N/m^{2}\right]$;
  - The useful volume (which also defines the degree of filling) of the piece $V_{util}\;\left[m^{3}\right]$.
    In this sense, combinations of variables are also allowed, such as:
  - The axial moment of inertia $I_{z}\;\left[m^{4}\right]$, if it is desired to replace the dimensions a [m],  b[m] and thereby attach a more flexible model (not necessarily a rectangular section!) to the studied prototype;
  - The stiffness module $E\cdot I_{z}\;\left[N\cdot m^{2}\right]$, if the original/traditional material used in the prototype is abandoned and only the size of their product $E\cdot I_{z}$ will matter, without imposing distinct restrictions on the material and the axial moment of inertia.
2. The creation of the matrix A (see Table 1), formed by the exponents of the dimensions of the variables considered to be independent, i.e., those variables, the size of which is chosen a priori independently, both in the prototype and in the model; this matrix must be invertible, i.e., det|A|≠0; with the help of this set of variables, particularly flexible models can be obtained, which will lead to as simple, cheap and repeatable experimental investigations as possible;
3. The creation of the matrix B, formed by the exponents of the dimensions of the variables considered to be dependent, i.e., those variables whose size is chosen a priori independently only for the prototype, while for the model, they will necessarily result only by applying an element of the *ML*, which is to be deduced; among these dependent variables is the vertical displacement sought at the prototype v [m], which will result exclusively by applying an element of the *ML* that will be deduced, depending on the displacement actually measured on the model;
4. To this set of matrices *B-A* are attached the matrices $C=-{\left(A^{-1}\cdot B\right)}^{T},$ respectively; $D\equiv I_{nxn}$, which, together with the matrices *A* and *B*, will constitute the Dimensional Set (*DS*) in the form

It is mentioned above that we have the inverse of the matrix *A*, that is $\left(A^{-1}\right)$, the transpose of the product $\left(A^{-1}\cdot B\right),$ and the number of lines from *A* and *B* being equal to the number of basic dimensions involved, while for *C* and *D*, we have the number *(n)* of the dimensionless variables $\pi_{n}$, which helps to fully define the *ML*, as will be illustrated next. The matrix *D* represents the appropriate unit matrix n×n.

5. A Scale factor
  (2) $$S_{\omega }\;\left[-\right]=\frac{\omega_{2}}{\omega_{1}},$$
  is defined for each variable ω (dependent, independent, respectively), where the index “1” refers to the prototype, and “2” to the model;
6. Based on a unique protocol [18,22,23,26,28,92,93],:
  - All elements are extracted, i.e., dimensionless variables $\pi_{j},\;\;j=1,\;\ldots ,\;n$, which will actually be products of independent variables at certain powers (results from Dimensional Set) and a dependent variable at the first power;
  - Each dimensionless variable obtained $\pi_{j}=1,\;\;j=1,\;\ldots ,\;n$ in this way is equal to unity;
  - The initial variables are replaced with their Scale Factors;
  - Each Scale Factor of the dependent variable from the respective equality will be expressed by $\pi_{j}=1,\;\;j=1,\;\ldots ,\;n$;
  - By applying that relationship $S_{\omega }\;\left[-\right]=\frac{\omega_{2}}{\omega_{1}}$, the desired size will finally result, which is further illustrated in the first relationships related to the different approaches.

Next, some of these *ML*, deduced according to other dependent variables, will be presented.

The first approach assumes that the independent variables (which can be freely chosen a priori for both the prototype and the model) are the longitudinal modulus of elasticity $E\;\left[N/m^{2}\right]$ and the axial moment of inertia $I_{z}\;\left[m^{4}\right]$, and the dependent variables are the rest of the variables. Within this approach, the authors also wanted to highlight the fact that the parameter $a^{*}\;\left[m\right]\;\Leftrightarrow \;a_{1},\;\;a_{2},\;\;b_{1},\;\;b_{2},\;\;b_{3},\;\;c_{1},\;\;d_{1},\;\;d_{2},\;\;e_{1},\;\;h_{1}$ can be divided into others, such as: $a^{*}\;\left[m\right]\;\Leftrightarrow \;a_{1},\;\;a_{2},\;\;e_{1}$; $b^{*}\;\left[m\right]\;\Leftrightarrow \;b_{1},\;\;b_{2},\;\;b_{3}$; $c^{*}\;\left[m\right]\;\Leftrightarrow \;c_{1},\;\;d_{1},\;\;d_{2},\;\;h_{1}$, without affecting the essence of the *ML*.

At the same time, in order to correctly merge the sizes a [m],  b[m] in $I_{z}\;\left[m^{4}\right]$, they (that is, a and b) can no longer appear in other elements, as they are here $a^{*},\;\;b^{*},\;\;c^{*}$.

The Dimensional Set had the following elements (Table 2):

By applying the unique protocol, the following resulted in turn:

- From the first line, i.e., of $\pi_{1}$, the exponents of the variables led to the following product, which was equal to unity, and subsequently led to the variables being substituted with their Scale Factors, finally resulting in the first *ML*:
  (3) $$\pi_{1}=v\cdot E^{0}\cdot I_{z}^{-0.25}=1\;\Rightarrow \;S_{v}=\sqrt[{4}]{S_{I_{z}}}.;$$
- From this law, based on the experimental measurements made on the model, the size $v_{2}$ is known, and consequently, from $S_{v}=\sqrt[{4}]{S_{I_{z}}}=\frac{v_{2}}{v_{1}}$ will finally result in the size of $v_{1}$, that is:
  (4) $$v_{1}=\frac{v_{2}}{\sqrt[{4}]{S_{I_{z}}}};$$
- The rest of the elements of the *ML* will be interpreted in a similar way, such as for example the one necessary to establish the size of the force applied to the model, knowing a certain amount of force, which would require the prototype:
  (5) $$\pi_{2}=F\cdot E^{-1}\cdot I_{z}^{-0.5}=1\;\Rightarrow \;S_{F}=S_{E}\cdot \sqrt{S_{I_{z}}};$$
  so:
  (6) $$F_{2}=F_{1}\cdot S_{E}\cdot \sqrt{S_{I_{z}}};\;\mathrm{etc}.$$

A second approach involved a merging of the modulus of elasticity $E\;\left[N/m^{2}\right]$ and the axial moment of inertia $I_{z}\;\left[m^{4}\right]$ into the new independent variable of the stiffness modulus $E\cdot I_{z}\;\left[N\cdot m^{2}\right]$, thus allowing both a more favorable choice of material type in combination with an equally preferable cross-section and the release of an independent variable, allowing the introduction of the length of the beams in this free place (thus the free choice of the two lengths in the prototype and in the model).

The Dimensional Set had the following elements (Table 3):

Following the application of the above protocol, the seven elements of the Model Law resulted:

(7) $$\pi_{1}=v\cdot L^{-1}\cdot {\left(E\cdot I_{z}\right)}^{0}=1\;\Rightarrow \;\frac{v}{L}=1\;\Rightarrow \;v=L\;\Rightarrow S_{v}=S_{L};$$

(8) $$\pi_{2}=a*\cdot L^{-1}\cdot {\left(E\cdot I_{z}\right)}^{0}=1\;\Rightarrow \;S_{a*}=S_{L};$$

(9) $$\pi_{3}=b*\cdot L^{-1}\cdot {\left(E\cdot I_{z}\right)}^{0}=1\;\Rightarrow \;S_{b*}=S_{L};$$

(10) $$\pi_{4}=c*\cdot L^{-1}\cdot {\left(E\cdot I_{z}\right)}^{0}=1\;\Rightarrow \;S_{c*}=S_{L};$$

(11) $$\pi_{5}=A_{1}\cdot L^{-2}\cdot {\left(E\cdot I_{z}\right)}^{0}=1\;\Rightarrow \;S_{A_{1}}={\left(S_{L}\right)}^{2};$$

(12) $$\pi_{6}=F\cdot L^{3}\cdot {\left(E\cdot I_{z}\right)}^{-1}=1\;\Rightarrow \;S_{F}=\frac{S_{EIz}}{{\left(S_{L}\right)}^{3}};$$

(13) $$\pi_{7}=V_{u}\cdot L^{-3}\cdot {\left(E\cdot I_{z}\right)}^{0}=1\;\Rightarrow \;S_{V_{u}}={\left(S_{L}\right)}^{3}.$$

Of these, for the concrete application, those related $v_{1}$ to and $F_{2}$, were of particular interest.

A third approach assumed the introduction as an independent variable of the useful volume $V_{util}\;\left[m^{3}\right]$ (therefore a parameter of the degree of filling) in addition to the stiffness modulus $E\cdot I_{z}\;\left[N\cdot m^{2}\right]$. Consequently, both the force applied to the model and its length resulted as dependent variables, thus obtained based on the *ML*. The *DS* had the following elements (Table 4):

In this case, only the elements related to the vertical displacement of the free end of the prototype $v_{1}$, the force that must be applied to the model $F_{2}$, and the required length of the model $L_{2}$ are presented, that is:

(14) $$\pi_{1}=v\cdot V_{u}^{-0.333}\cdot {\left(E\cdot I_{z}\right)}^{0}=\frac{v}{\sqrt[{3}]{V_{u}}}=1\;\Rightarrow \;S_{v}=\sqrt[{3}]{S_{V_{u}}}.$$

(15) $$\pi_{6}=F\cdot V_{u}^{0.666}\cdot {\left(E\cdot I_{z}\right)}^{-1}=\frac{F\cdot \sqrt[{3}]{V_{u}^{2}}}{E\cdot I_{z}}=1\;\Rightarrow S_{F}=\frac{S_{EI_{z}}}{\sqrt[{3}]{{\left(S_{V_{u}}\right)}^{2}}}.$$

(16) $$\pi_{7}=L\cdot V_{u}^{-0.333}\cdot {\left(E\cdot I_{z}\right)}^{0}=\frac{L}{\sqrt[{3}]{V_{u}}}=1\;\Rightarrow \;S_{L}=\sqrt[{3}]{S_{V_{u}}}.$$

The following paragraph presents the way to obtain the desired values, including displacement of the free end of the prototype $v_{1}$.

## 3. Experimental Validation of the ML

To present the experimental validation of the Model Law, a prototype and a model in the form of cantilevers (beams embedded at one end and loaded at the free end with a vertically concentrated force) of solid rectangular sections were chosen, as in Figure 1, but placed on the small sides of the rectangle (in order to obtain greater stiffness).

The material was the usual plastic PLA (Polylactic Acid), the machine (the 3D printer) was of the Creality Ender-3 type, and the software used was Cura [94].

It was proposed to determine, with the help of the *ML* from case b), the displacement of the free end of the length $L_{1}=0.400\;m$ cross-section prototype 0.018×0.030 m under the action of the force $F_{1}=0.491\;N$, if the attached model was of length $L_{2}=0.300\;m$ and cross-section 0.010×0.020 m.

The axial moments of inertia are determined

(17) $$I_{z_{1}}=\frac{0.018\cdot {\left(0.030\right)}^{3}}{12}=40.05\cdot 10^{-9}\;m^{4};I_{z_{2}}=\frac{0.010\cdot {\left(0.020\right)}^{3}}{12}=6.67\cdot 10^{-9}\;m^{4}.$$

We have $E_{1}=E_{2}=2.31\cdot 10^{9}\;\frac{N}{m^{2}}$, and the scale factor results:

$$S_{E}=\frac{E_{2}}{E_{1}}=1\;$$

and therefore, $S_{E\cdot I_{z}}=S_{I_{z}}=0.167$.

Based on the lengths (chosen a priori independently), it results that $S_{L}=\frac{L_{2}}{L_{1}}=0.75$.

Thus, from the sixth element of the *ML*, i.e.,

$$\pi_{6}=F\cdot L^{3}\cdot {\left(E\cdot I_{z}\right)}^{-1}=1\;\Rightarrow \;S_{F}=\frac{S_{EIz}}{{\left(S_{L}\right)}^{3}}$$

they will result in turn the magnitude of force *F*_2_:

(18) $$S_{F}=\frac{S_{EIz}}{{\left(S_{L}\right)}^{3}}=\frac{F_{2}}{F_{1}}\;\Rightarrow \;\;F_{2}=F_{1}\cdot \frac{S_{EIz}}{{\left(S_{L}\right)}^{3}}=0.491\cdot \frac{0.167}{{\left(0.75\right)}^{3}}=0.203\;N.$$

Carrying out the measurements on the model with the force $F_{2}$, the displacement $v_{2}=0.000583\;m$ resulted and from the first element of the *ML*

$$\pi_{1}=v\cdot L^{-1}\cdot {\left(E\cdot I_{z}\right)}^{0}=1\;\Rightarrow \;S_{v}=S_{L}$$

we have obtained the predictable displacement of the prototype $v_{1,p}$:

(19) $$S_{v}=S_{L}=\frac{v_{2}}{v_{1}}\Rightarrow \;v_{1}=\frac{v_{2}}{S_{L}}=\frac{0.000583}{0.75}=0.000778\;m.$$

After carrying out actual measurements on the prototype, $v_{1\;M}=0.000786\;m$ was obtained, so an error of 1.08 %, with respect to the predictable value $v_{1,p}$, which is acceptable from an engineering point of view.

Consequently, the *ML* provided by version (b) is valid for the analyzed case.

## 4. Discussions

1. It could be noted that *MDA* presents great flexibility, and thus, the model can be made in optimal conditions (manufacturing, testing, number and qualification of personnel, etc.);
2. Addition represents a very promising current trend, and the implementation of *MDA* in the design of models, which will facilitate the creation of the final product, i.e., prototypes, represents an area that deserves to be deepened.
3. Elements of the *ML* are not proper physical relations in the usual sense; they represent only correlations between the scale factors of the variables involved in describing the behavior of the prototype in relation to that of the model;
4. Depending on the purpose of new models, it will be possible to apply the other two laws of the model deduced and analyzed in Section 2;
5. If an emphasis will be placed on the involvement of different materials for the model and prototype, or/and on the use of different cross-sections related to them, then the different degrees of filling can be explored as an optimization parameter (through the variables $E\cdot I_{z}$, as well as $V_{util}$);
6. If different degrees of filling will be imposed (with the help of $V_{util}$), or/and stiffness modules chosen a priori, then by means of the dependent variables $\left(a^{*},\;\;b^{*},\;\;c^{*}\right)$ and $A_{1}$, respectively, new solutions can be found, with new forms of stiffening of the cross section, respectively, the length of the model involved in experimental investigations.

## 5. Conclusions

The advantages of MDA can be summarized as follows:

- It does not require deep knowledge in the field, only the review of all variables, which can to a certain extent influence the studied phenomenon;
- Insignificant variables (either from a physical point of view, or from the point of view of the weight in the unfolding of the respective physical phenomenon) are automatically eliminated by the protocol developed by Szirtes [16,17];
- The methodology is unitary, easy and allows obtaining all the dimensionless variables related to the phenomenon, so it provides the complete version of the Model Law (*ML*), which can be achieved through the previously mentioned methods only in completely and completely particular cases;
- *MDA* is a flexible method, as was illustrated in Section 3, allowing us, from the approach of the general case, to obtain a series of particular versions without any difficulty;
- The *MDA* protocol allows the grouping of variables in an optimal manner, i.e., to highlight those variables, which depend on the testing of the model under optimal conditions and thus obtain the most reliable and repeatable information that will allow the description of the behavior of the analyzed prototype.

Among the future objectives of the authors is the expansion of these theoretical and experimental investigations, which would make available to specialists new laws of the model, developed with the help of *MDA*, which would be able to take into account other important aspects of the additive manufacturing of complex parts, having internal structures different from the current ones.

The *ML* deduced in Section 2 and validated in Section 3 serves as an illustration of how *MDA* can provide solutions for testing models at a low cost price and under rigorously reproducible conditions in order to complete the intended real product, i.e., the prototype, an example serving to validate the proposed method in polymers additive manufacturing optimization.

As seen, the presented *MDA* methodology is unitary, simple and safe and accessible to any researcher and can represent an effective way to optimize the finished products made with *AM* technology.

Taking into account the importance of *AM* in practice, numerous works have addressed different aspects identified in the study of this procedure. *AM* allows the use in different industrial branches, allowing the manufacturing of materials.

The importance of the work lies in the fact that it demonstrates the possibility of applicability in *AM* a field that is at the beginning of its development and which, alone, will come with numerous problems that can be solved with the method proposed and validated in the work.

## Figures and Tables

**Table 1:** Dimensional Set.

***B***	***A***
***D***	***C***

**Table 2:** First approach. The explicit Dimensional Set.

Dimensions	B								A	
	v	F	L	a*	b*	c*	A_1_	V_util_	E	I_z_
m	1	0	1	1	1	1	2	3	−2	4
N	0	1	0	0	0	0	0	0	1	0
π1	1	0	0	0	0	0	0	0	0	−0.25
π2	0	1	0	0	0	0	0	0	−1	−0.5
π3	0	0	1	0	0	0	0	0	0	−0.25
π4	0	0	0	1	0	0	0	0	0	−0.25
π5	0	0	0	0	1	0	0	0	0	−0.25
π6	0	0	0	0	0	1	0	0	0	−0.25
π7	0	0	0	0	0	0	1	0	0	−0.5
π8	0	0	0	0	0	0	0	1	0	−0.75

**Table 3:** Second approach. Dimensional Set.

Dimensions	B							A	
	v	a*	b*	c*	A_1_	F	V_util_	L	E × I_z_
m	1	1	1	1	2	0	3	1	2
N	0	0	0	0	0	1	0	0	1
π1	1	0	0	0	0	0	0	−1	0
π2	0	1	0	0	0	0	0	−1	0
π3	0	0	1	0	0	0	0	−1	0
π4	0	0	0	1	0	0	0	−1	0
π5	0	0	0	0	1	0	0	−2	0
π6	0	0	0	0	0	1	0	3	−1
π7	0	0	0	0	0	0	1	−3	0

**Table 4:** Third approach. New Dimensional Set.

Dimensions	B							A	
	v	a*	b*	c*	A_1_	F	L	V_util_	E × I_z_
m	1	1	1	1	2	0	1	3	2
N	0	0	0	0	0	1	0	0	1
π1	1	0	0	0	0	0	0	−0.33333	0
π2	0	1	0	0	0	0	0	−0.33333	0
π3	0	0	1	0	0	0	0	−0.33333	0
π4	0	0	0	1	0	0	0	−0.33333	0
π5	0	0	0	0	1	0	0	−0.66667	0
π6	0	0	0	0	0	1	0	0.666667	−1
π7	0	0	0	0	0	0	1	−0.33333	0

## Data Availability

Not applicable.

## Nomenclature

*A*	Area (m^2^);
*F*	Force (N);
*g*	Gravitational acceleration (m/s^2^);
*a, b, a*, b*, c*, l, L*	Length (m);
*V*	volume (m^3^);
*w*	velocity (m/s);
*v*	vertical displacement (m)
$S_{\dot{Q}}\;,\;\;S_{L_{z}}\;,\;\;S_{\Delta t}\;,\;\;S_{\tau }\;,\;\;S_{\lambda_{x\;steel}}\;,S_{\varsigma }$ $S_{F}\;,\;\;S_{L}\;,\;\;S_{E}\;,\;\;S_{E\cdot I_{z}},\;\;S_{v}$	scale factor corresponding to the sizes indicated in the index.
Greek symbols	
ρ	Density (kg/m^3^);
∇ $\pi_{j},\;\;j=1,\;\ldots ,\;n$ γ	Nabla operator; Dimensionless variables; Specific gravity (N/m^3^).
Subscripts	
*x, y, z*	Directions.
